# New or little-known species of the genus *Amphimenes* Bates, 1873 (Coleoptera, Carabidae, Lebiinae) from Vietnam

**DOI:** 10.3897/zookeys.65.503

**Published:** 2010-10-29

**Authors:** Dmitry N. Fedorenko

**Affiliations:** Amphimenes N. Severtsov Institute of Ecology and Evolution, Russian Academy of Sciences, Moscow, Russia

**Keywords:** new species, species group, new combination, Amphimenes, Brachichila, Vietnam

## Abstract

Twelve new species of the genus Amphimenes Bates, 1873 are described from Vietnam: Amphimenes maculatus **sp. n.**, Amphimenes bidoupensis **sp. n.**, Amphimenes gracilis **sp. n.**, Amphimenes montanus **sp. n.**, Amphimenes giganteus **sp. n.**, Amphimenes medius **sp. n.**, Amphimenes minutus **sp. n.**, Amphimenes rufipes **sp. n.**, Amphimenes reflexicollis **sp. n.**, Amphimenes planicollis **sp. n.**, Amphimenes nitidus **sp. n.**, and Amphimenes kabakovi **sp. n.**. Amphimenes rugulipennis Bates, 1892, **comb. n.**, is transferred from the genus Brachichila Chaudoir, 1869 and is redescribed from fresh material. A key to all congeners is provided, these being arranged into five new species-groups. Adults of the *rugulipennis*- and *piceolus*-groups show subcortical habits, while those of the *medius*-, *rufipes-* and *planicollis*- groupsare herpetobiotic, resulting in such morphological adaptations as partly reduced eyes, missing wings and adnate elytra.

## Introduction

The genus Amphimenes Bates, 1873 is still a poorly-known member of the subtribe Pericalina, Lebiini. It was established for the only, and type, species Amphimenes piceolus Bates, 1873 from Japan (Bates 1873). Two further congeners, Amphimenes asahinai Nakane, 1957, and Amphimenes ryukiuensis Habu, 1964, were added from Taiwan and Japan (Ryukyus; Amamioshima Is., Kyushu), respectively, while the genus was revised by (Habu (1964, 1967). He reviewed and keyed all these species, and pointed out that the earlier records of Amphimenes piceolus in Taiwan and Fujian [Fukien], southern China (Jedlička 1940, 1953, 1963) actually belonged to different species. In addition, Amphimenes rugulipennis (Bates 1892), comb. n., has hitherto been considered as a member of the genus Brachichila Chaudoir, 1869, this being corrected here.

Among the other members of Oriental Pericalina, Amphimenes seems to be especially similar to the genera Brachichila and Dolichoctis Schmidt-Goebel, 1846, differing well from both chiefly by the combination of a well-developed median tooth on the mentum, a highly characteristic cross-striation on the elytra and some other characters. The adults of at least some winged species (Amphimenes piceolus) have been known to live under the bark [of dead trees] (Habu 1964, 1967).

During a few recent expeditions of the Russia-Vietnam Tropical Center to Vietnam, I collected eight species of the genus, all of them but Brachichila rugulipennis being new. Only three of these new species showed subcortical habits and well-developed wings, while the remaining ones dwelled under or in logs or larger branches on the soil surface and were wingless following their habits. This proved to also be true of four species of five further described here as new, these collected by O. N. Kabakov in Vietnam five decades ago. As a result, all congeners are keyed here, with the Vietnamese species described or redescribed. Taking new characters into account, I also think it advisable now to refine the diagnosis of the genus.

One to 18 specimens per species studied were measured concerning the following parameters: body length from the apex of the mandible to the apex of the abdomen/elytra, head width across eyes (HW), maximum pronotal and elytral widths (PW and EW, respectively), length of pronotum along its mid-line (PL), length (MESL) and width (MESW) measured along the outer and anterior margin of the metepisternum, respectively, length from the basal margin to the apex of the elytra (EL), and distances between the elytral basal margin and the discal setigerous pores (D1, D2 and D3). The indices PW/HW, PW/PL, EL/EW, EW/PW, MESL/[MES]W, as well as D1/EL, D2/EL and D3/EL, were analysed.

Holotypes and paratypes of the species described here are deposited in the Zoological Institute of the Russian Academy of Sciences, St. Petersburg (ZISP), Zoological Museum of the Moscow State University (ZMMU), and the author’s reference collection at the A. N. Severtsov Institute of Ecology and Evolution, Russian Academy of Sciences (SIEE), as indicated hereafter.

## Taxonomy

### The Genus Amphimenes

Bates, 1873

Amphimenes piceolus  Bates, 1873: 322 (Nagasaki, Japan; by monotypy) [Type-species].

#### Redescription.

Small- to medium-sized pericaline lebiines, either unicoloured or with a pale pattern composed of two, posthumeral and subapical, rounded, yellow spots on each elytron, latter spots adjoining suture; reflexed side margins of both pronotum and elytra, antennae, mouthparts, legs, labrum and clypeus usually paler, often contrastingly so. Body glabrous, except for underside microscopically ciliate and pronotum almost indistinctly so in some species. Forebody dorsum dull due to an almost granulate isodiametric microsculpture occupying head, pronotum and scutellum, sometimes somewhat shining because of a weaker microsculpture which, in addition, forms slightly transverse meshes behind pronotal front margin and base. Elytral microsculpture composed of very fine and dense transverse lines or very narrow transverse meshes, anyway contributing to dorsum’s iridescence, rarely coarse and isodiametric. All but one congener show a highly characteristic cross-striated sculpture occupying entire disc or, rarely, only elytral base; this cross-striation, combined with microsculpticells or lines, becoming increasingly oblique outwards, resulting in both being conspicuously oblique posterolaterad on intervals 5 to 8.

**Figures 1–3.**
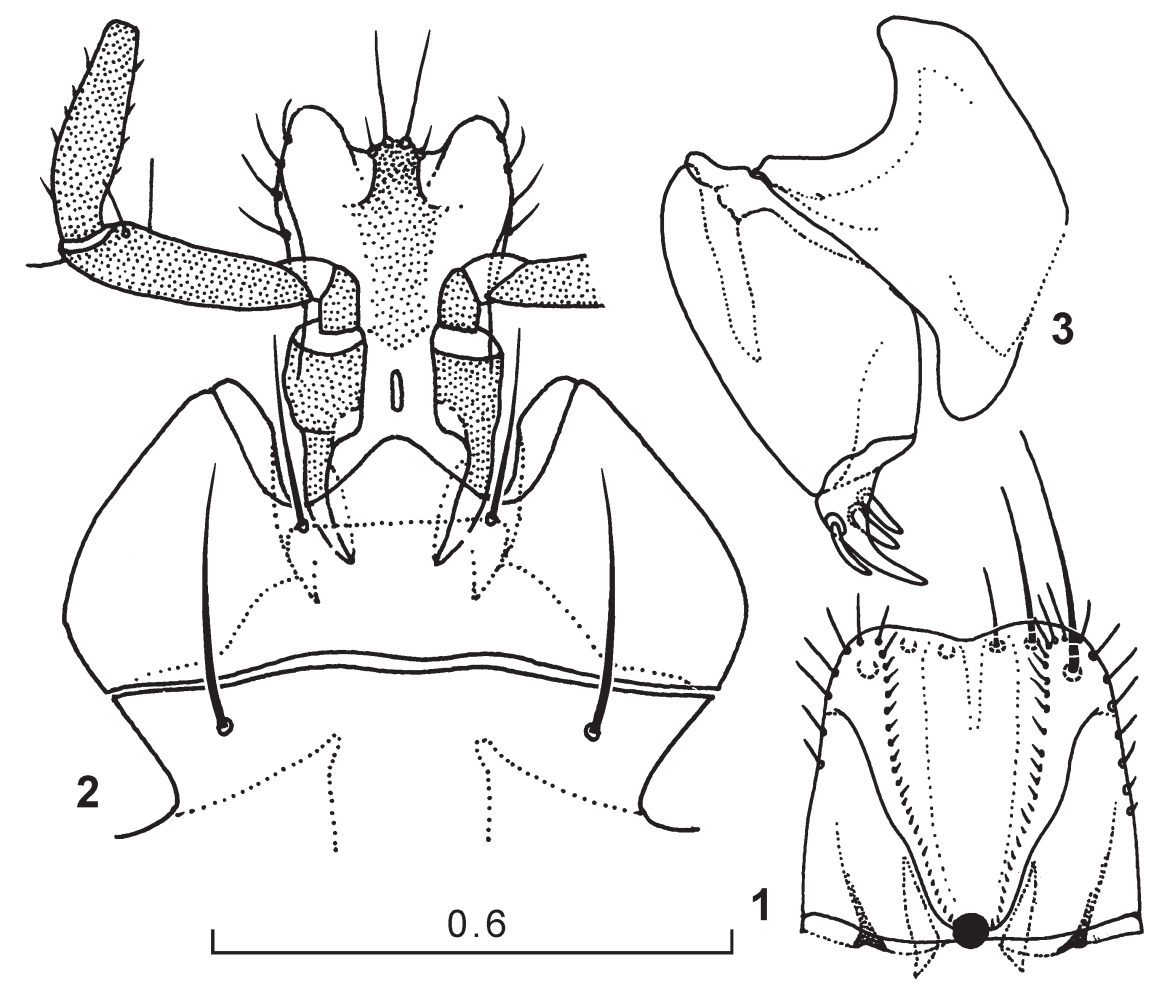
Genus Amphimenes, ventral aspect: labrum (**1**), labium (**2**), left gonapophyse (**3**).

Eyes varying from large and hemispherical to small and flat. Labrum trapezoidal (Fig. 1), a little narrowing forwards, with front angles rounded; anterior margin slightly sinuate, with six setae of gradually decreasing length inwards. Last maxillary palpomere narrowing apicad, longer than penultimate one. Submentum with a pair of strong setae; another pair situated at base of a large median tooth of mentum, latter rounded apically, about half as long as wide lateral lobes (Fig. 2). Ligula well-developed, sclerotized apically and not so strongly basally, fairly narrow, with two pairs of apical setae, inner much longer than outer; paraglossae membranous, wide, a little longer than ligula, each with four setae over outer margin. Penultimate labial palpomere with two preapical setae drawn together; proximal and distal setae of anterodorsal and anteroventral position, respectively; last labial palpomere subcylindrical, slightly narrowing apicad. Antennae long to short, pubescent from apical half of antennomere 4 onward, some species (Amphimenes medius, Amphimenes minutus, Amphimenes rufipes) showing apical half of antennomere 3 spa s ly ciliate along outer margin; 3rd antennomere 1.2–2.05 times as long as 2nd; 7th to 9th 1.22–3.34 times as long as wide.

Pronotum rather flat, strongly transverse to about as long as wide, weakly cordate, deeply emarginate anteriorly, with protruding and fairly narrow front angles, rather strongly rounded on sides, somewhat narrowing basad, broadest before middle, *i.e.* level to anterolateral setigerous pore at which side margin often slightly angulate; latter moderately strongly sinuate to straight before posterior angles, these varying from obtuse to almost indistinct due to pronotal base increasingly oblique forward towards posterolateral setigerous pore. Front margin with a very narrow but distinct polished bead; side margin rather strongly explanate-reflexed, narrow anteriorly and gradually broadening basad; basal transverse depression conspicuous to weak, when well-developed, ca 0.15 times as long as pronotum along mid-line, latter varying from deep to superficial; lateral basal foveae weak to indistinct, each usually extended to the middle or anterior third as a very shallow depression running parallel to side margin; disc at middle with a pair of small and shallow paramedian foveae. Base slightly trilobed, with medial part mostly a little surpassing lateral lobes.

Metathorax longer in winged than in wingless species; accordingly, metepisternum much longer than wide (MESL/W=1.35–1.73) in the former versus about as long as wide (MESL/W=0.78–1.02) in the latter.

Elytra subrectangular to oval, subparallel to rather strongly rounded on sides, with base wide to very narrow and shoulders slightly to completely rounded; apical truncature oblique and slightly sinuate between outer angle of each elytron and its apex, those rounded in all but one species. Striae impunctate, deep throughout, intervals mostly convex. Discal setigerous pores three or two (either D1+D3 or D2+D3), adjoining stria 3 (D1) or 2 (D2, D3). Prescutellary pore present. Preapical setigerous pores (those of 7th interval) two, outer (anterior) large, inner (posterior) small. Umbilicate series uninterrupted or divided into two, posthumeral and subapical, groups.

Profemur rather strong. Claws serrate in basal two-thirds. Pretarsus with two or only one, distal, pair of setae beneath.

Aedeagus. Penis mostly rather stout, weakly arcuate, strongly bent to the right just behind basal ca s le and often twisted to the right behind the middle, with apical orifice rounded and shifted to the left; apical lamella rather small, of characteristic shape and structure in different species. Microsculpture usually conspicuous, composed of isodiametric to slightly longitudinal meshes predominantly developed over left side; right ventral side mostly longitudinally striate in basal half. Parameres moderately strongly reduced, left paramere subrectangular, right one small, rounded apically, with base long, narrow and hooked apically.

Female gonapophyse as in Fig. 3.

Sexual dimorphism: male protarsomeres 1–3 dilated, each with an adhesive pubescence beneath, sternite 6 sometimes slightly desclerotized or weakly emarginate posteriorly, with one, lateral, pair of setae, versus not sinuate and with two pairs of setae in female. Males of two species show basal third of profemur underside furnished with a small tubercle instead of a pointed mesotrochanteral tubercle observed in males of most other congeners.

#### Geographic distribution.

The range of the genus extends from Japan (Kyushu and Shikoku) in the north and east to at least southern Vietnam in the south, and Myanmar in the west. Winged congeners are certain to be much more widespread than wingless ones, implying still undescribed species of very local ranges to be discovered in unexplored montane regions of Indochina, Vietnam in particular.

#### Habits and habitats.

All congeners inhabit forested areas, both montane and lowland. Original adult habits seem to be strictly subcortical. In southern Vietnam, species with such habits occur under bark (Amphimenes bidoupensis) or also in chapped bark of standing dead trees (Amphimenes rugulipennis, Amphimenes maculatus), never of fallen deadwood or logs. All of them are winged, some flying to light at night. Such soil-dwellers as Amphimenes medius, Amphimenes minutus, Amphimenes rufipes and Amphimenes giganteus are certain to be derived, being adapted to living in leaf litter or cavities in logs or fallen larger branches. This has resulted in a few morphological adaptations, among them, the hindwings missing, the elytra wide, fused along the suture, with strongly rounded shoulders, while the eyes tending to be reduced to small and flat. The latter contributes much to the head being narrower across the eyes while broader across the neck, all relative to body width. Only Amphimenes giganteus among the members of the latter ecological group sometimes occurs under bark, while the others have never been observed there. Four species from northern Vietnam show similar morphological adaptaptions, suggesting herpetobiotic habits as well.

In the Bi Doup – Nui Ba Nature Reserve, three to five species live sympatrically, with up to four soil-dwellers among them occurring syntopically. This seems to also be true for elsewhere in Vietnam north of the Dalat Plateau because more than one species have been recorded from such localities as Tam Dao (Amphimenes reflexicollis, Amphimenes kabakovi), Quỳ Châu (Amphimenes gracilis, Amphimenes planicollis) or Thai Nguen (Amphimenes gracilis, Amphimenes nitidus).

#### Generic composition.

The genus currently includes 16 species arranged into five species groups. Of them, two, the *rugulipennis*-group (Amphimenes rugulipennis, Amphimenes maculatus) and the *piceolus*-group (Amphimenes piceolus, Amphimenes riukyuensis, Amphimenes montanus, Amphimenes bidoupensis, Amphimenes gracilis, perhaps also Amphimenes asahinai) comprise congeners of subcortical habits. The *medius*-group (Amphimenes giganteus, Amphimenes medius and Amphimenes minutus) and the *planicollis*-group (Amphimenes planicollis, Amphimenes reflexicollis, Amphimenes nitidus and Amphimenes kabakovi) are established here for soil-dwelling species distinguishable chifly in a particular formula of the elytral discal chaetome. This is also true of Amphimenes rufipes which seems to constitute a group of itself.

#### Key to species of Amphimenes

**Table:** 

1(2)	Elytra without distinct cross-striation but with a coarse isodiametric microsculpture on disc. Body small, 5.0 mm long, unicoloured, with appendages contrastingly paler. Eyes small and flattened. Elytra broadest behind the middle, D2+D3, D2 situated at middle. Wings absent. Antennae very short	9. Amphimenes rufipes sp. n.
2(1)	At least elytral base with a distinct cross-striation, elytral microsculpture composed of strongly transverse meshes or transverse lines not forming distinct meshes. Coloration other than above.	
3(6)	Elytra dark, with a pale spotted pattern (Figs 4, 5). Body appendages yellow, contrastingly paler than body dorsum. D1 (near base, D1/EL = ca 0.15) + D2 (at middle) + D3. Pronotum transverse, 1.51–1.60 times as wide as long. Wings full.	
4(5)	Larger, 6.4–8.2 mm long. Elytral microsculpture composed of fine and very dense transverse lines not forming distinct meshes. Elytral paler spots smaller, anterior spot extended to shoulder while not surpassing the midway between D1 and D2; posterior spot not extending forward beyond the midway between D2 and D3. Male profemur ventrally with a small tubercle in basal third	1. Amphimenes rugulipennis
5(4)	Smaller, 5.6–5.9 mm long. Elytral microsculpture consisting of strongly transverse but distinct meshes. Paler spots on elytra larger, anterior spot not extending towards shoulder laterally while surpassing the midway between D1 and D2; posterior spot extending forward beyond the midway between D2 and D3. Male profemur ventrally without tubercle	2. Amphimenes maculatus sp. n.
6(3)	Elytra monochromous dark, body appendages mostly darker than above. Discal setigerous pores two, D1+D3 or D2+D3, if three, then D2 situated far behind middle (D2/EL = 0.61–0.76).	
7(20)	D1+D2+D3.	
8(19)	D1 remote from elytral base (D1/EL = ca 0.25). Smaller, under 8.7 mm long, and predominantly winged species, with metepisternum distinctly longer than wide. Eyes either large and hemispherical or only slightly flattened; posterior supraorbital seta situated level to or slightly behind eye back margin.	
9(12)	Elytral microsculpture composed of transverse meshes. Body length 5.8–7.0/5.5–6.8 mm.	
10(11)	Wings full, both metepisternum and elytra longer, ca 1.4–1.5 times as long as wide, latter less strongly rounded on sides (ex Habu, 1964)	Amphimenes piceolus Bates, 1873
11(10)	Wings vestigial, metepisterna and elytra shorter, MESL/W=1.07–1.11, EL/EW=1.33; elytra more strongly rounded on sides (ex Habu, 1964)	Amphimenes asahinai Nakane, 1957
12(9)	Elytral microsculpture consisting of fine and dense transverse lines not forming meshes. Wings full.	
13(16)	Pronotum subcordate, with sides distinctly sinuate before posterior angles.	
14(15)	On average, larger, 6.7–8.0/7.1–8.3 mm long. Pronotum short and wide, 1.36–1.55 (mean 1.44) times as wide as long. Metepisternum long, 1.55–1.73 times as long as wide. Elytra with moderately dense and deep cross-striation and middle elytral intervals nearly flat behind the middle	3. Amphimenes bidoupensis sp. n.
15(14)	On average, smaller, 6.4–7.5 mm long. Pronotum narrower and longer, 1.29–1.43 (mean 1.37) times as wide as long. Metepisternum shorter, 1.35–1.43 times as long as wide. Elytra with very dense and deep cross-striation and intervals more or less convex behind the middle	4. Amphimenes gracilis sp. n.
16(13)	Sides of pronotum not or indistinctly sinuate before posterior angles.	
17(18)	Pronotum weakly narrowing basad, broader relative to head (PW/HW=1.49–1.57), with reflexed side margin basally broader than in the following species. Elytra and metepisternum shorter, EL/EW=1.43–1.50, MESL/W=1.38–1.42. Body colour darker, femora infuscate except at apices	5. Amphimenes montanus sp. n.
18(17)	Both pronotum and its side margin narrower basad, and PW/HW≤1.4. Elytra and metepisternum longer, EL/EW over 1.5, MESL/W over 1.6. Body 6.8–7.5/6.5–7.1 mm long, slightly paler, with legs monochromous reddish-yellow.	Amphimenes ryukyuensis Habu, 1964
19(8)	D1 close to base (D1/EL = ca 0.13). Large, 8.5–10.6 mm long, and wingless. Metepisterna short. Elytra strongly and regularly rounded on sides and rather strongly sinuate between a pointed acute apex and a protruding outer angle of apical truncature. Pronotum long, 1.18–1.26 (mean 1.22) times as wide as long. Eyes fairly small and distinctly flattened, posterior supraorbital seta situated level to about 1/3 distance between eye back margin and pronotal front margin. Antennae long. Body black	6. Amphimenes giganteus sp. n.
20(7)	Either D1+D3 or D2+D3. Wingless, both metepisternum and elytra short, latter with strongly rounded shoulders and sides.	
21(24)	D1 (near base) + D3. Eyes slightly to strongly reduced.	
22(23)	Larger and stouter, 5.7–7.2 mm long. Eyes less strongly reduced, posterior supraorbital seta situated level to about 1/3 distance between eye back margin and pronotal front margin. Antennae longer, surpassing base of pronotum	7. Amphimenes medius sp. n.
23(22)	Smaller and slenderer, 5.3–6.3 mm long. Eyes very small and flat, posterior supraorbital seta situated level to about midway between eye back margin and pronotal front margin. Antennae not reaching pronotal base	8. Amphimenes minutus sp. n.
24(21)	D2+D3. Eyes slightly reduced.	
25(28)	Elytra conspicuously cross-striated throughout, D2 situated mostly behind the middle (D2/EL=0.55–0.67).	
26(27)	Pronotum with sides distinctly sinuate before posterior angles, a moderately wide and impunctate reflexed side margin and an almost straight base, but for posterior angles oblique forward. Body appendages reddish-yellow. Smaller, 5.4–6.6 mm long	11. Amphimenes planicollis sp. n.
27(26)	Sides of pronotum indistinctly sinuate before posterior angles, side margin strongly reflexed, very wide basally, finely but distinctly rugulose-punctate; base with lateral lobes oblique posterodistad, thus surpassing its middle part. Femora infuscate except at apices. Larger, 8.5 mm long 10.	Amphimenes reflexicollis sp. n.
28(25)	Cross-striated sculpture fine and restricted to elytral base only, D2 situated before middle (D2/EL=0.41–0.44). Sides of pronotum not sinuate before posterior angles.	
29(30)	Elytral microsculpture composed of strongly transverse but distinct meshes. Pronotum rather narrow, 1.34 times as wide as long, 1.7 times as narrow as elytra, with posterior angles strongly oblique forward and thus almost indistinct. Eyes reduced in size and somewhat flattened. Body length 6.6 mm	13. Amphimenes kabakovi sp. n.
30(29)	Elytral microsculpture consisting of fine and dense transverse lines not forming meshes. Pronotum broader, 1.54 times as wide as long, 1.57 times as narrow as elytra, with posterior angles only slightly oblique forward. Eyes less reduced. Body larger, 7.5 mm long	12. Amphimenes nitidus sp. n.

## The *rugulipennis*-group

Elytra with paler spots; body colour sharply contrasting: dorsum black, body appendages, especially legs, elytral spots, as well as reflexed side margins of both pronotum and elytra reddish yellow. Body stout, with short elytra and strongly transverse pronotum (EL/EW=1.28–1.42, PW/PL=1.51–1.63). Discal setigerous pores on elytra three, both D1 and D2 being in anterior position (D1/EL=0.13–0.16, D2/EL=0.43–0.53).

Setigerous pores of umbilicate series divided into two, widely separated groups, posthumeral and preapical ones. Wings full. Last tarsomere with two, proximal and distal, pairs of ventral setae in all legs. Pronotum spa s ly but distinctly ciliate. Antennae moderately long, surpassing pronotal base. Eyes large hemispherical, posterior supraorbital seta situated level to eye back margin; tempora short and abruptly extending into neck. Frontal foveae not deep.

The group includes two species.

### 1. Amphimenes rugulipennis

(Bates, 1892) comb. n.

Figs 4 17 26 35

Brachichila ; Burma [Bates, 1892: 406].

#### Redescription.

Body length 6.4–8.2/6.3–7.7 mm, width 2.7–3.5 mm. Dorsum black, head dark brown to black; reflexed side margins of both pronotum and elytra, legs, mouthparts and antennae reddish yellow. Underside dark brown; gula, prosternum, median part of mesoventrite, metaventrite and abdomen red, with abdominal sternites 4–6 darkened laterally as well as 6 in apical half. Epipleura mostly dark, brown to dark brown. Elytron with two, rounded, yellow spots, isolated both from each other and elytral margin. Of them, anterior spot usually larger, occupying intervals 3 to 8, laterally almost extending to shoulder, mostly not surpassing midway between D1 and D2; posterior spot occupying four inner intervals and not extending forward midway between D2 and D3. Left and right posterior spots widely adjoining, thus often merging into a common macula sinuate along suture posteriorly.

3rd antennomere 1.58–1.76 (mean 1.65) times as long as 2nd, 8th 2.0–2.63 (mean 2.26) times as long as wide.

Pronotum 1.51–1.60 (mean 1.56) times as wide as long, 1.40–1.48 (mean 1.44) times as wide as head, fairly strongly rounded laterally, broadest before middle, a little narrowing basad, distinctly but not strongly sinuate before hind angles, latter obtuse due to base increasingly oblique forward at extremities. Anterior margin strongly sinuate between strongly protruding and apically rounded front angles. Base medial part distinctly convex backward, with a narrow reflexed border almost reaching hind angles. Mid-line deep, deeper basad, not adjoining anterior bead and abruptly disappearing before transverse basal depression, latter sublinear and not deep; lateral basal foveae weak; paramedian foveae small and very shallow. Reflexed side margin indistinctly separated from disc convexity by a narrow flat gutter.

Elytra broadly oval, 1.28–1.41 (mean 1.33) times as long as wide, 1.53–1.65 (mean 1.59) times as wide as pronotum, slightly rounded on sides, broadest in posterior third, with base wide, straight and transverse or slightly oblique towards widely rounded shoulders, apical truncature slightly sinuate and apices rounded narrowly and separately each. Elytral striae deep throughout, intervals more or less convex and subequally wide across disc; cross-striation rather fine, especially behind the middle. D1 at front border of anterior paler spot (D1/EL=0.12–0.16, mean 0.14), D2/EL=0.45–0.53 (mean 0.49), D3 at caudal border of posterior paler spot (D3/EL=0.86–0.92, mean 0.90). Metepisternum 1.46–1.50 times as long as wide.

Male profemur ventrally with a small but sharp tubercle in basal third.

Penis (Figs 17, 26, 35) regularly arcuate, microsculpture very coarse, apical orifice bilobed due to a strongly sclerotized dorsolateral projection extending to about its middle. Apical lamella small and triangular in lateral view, with a small rounded membranous window on right dorsal side.

**Figures 4–5.**
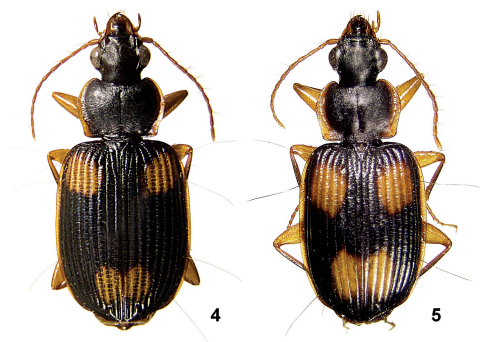
Amphimenes spp.: Amphimenes rugulipennis (**4**) and Amphimenes maculatus sp. n. (**5**).

#### Material.

Two syntypes (Museo Civico di Storia Naturale “Giacomo Doria”, Genova – MSNG), both ♂♂, labelled: “Tenasserim / Thagata / Fea. Apr. 1887” “SYNTYPUS” “Museo Civico di Genova”. The specimen is here designated as lectotype that bears additional labels: “Typus” “*rugulipennis* Bates” “Brachichila rugulipennis (es. tip.) Bates” “Brachichila rugulipennis Bates” “Brachichila rugulipennis Bates, 1892”.

Other material: 10 ♂♂, 4 ♀♀ (SIEE), South **Vietnam**, N[orthernmost part] of Dongnai Province, Nam Cat Tien National Park, 20–26.X. and 21–22.XI.2004 (D Fedorenko); ♀ (ZISP), Vietnam, Nghe An Prov., mountains NE of Cua Rao, 600 m a s l, 1.10.1962 (O N Kabakov); ♀ (ZISP), **Thailand**, Nakhon Prov., Ratchasima, env. of Khao Yai Natn. Park, 500–1000 m a s l, 26.X–4.XI.2000 (A Gorochov & L Anisyutkin).

#### Geographic distribution.

Myanmar, Thailand, southern Vietnam. Probably also Laos and Cambodia.

### 2. Amphimenes maculatus

Fedorenko sp. n.

urn:lsid:zoobank.org:act:E3F49490-F191-4939-9319-BD83B98E0A2D

Figs 5 18 27 36 

#### Description.

Very similar to the preceding species, except as follows: Body smaller, 5.6–5.9/5.3–5.7 mm long, 2.3–2.5 mm wide. Paler side margin of pronotum broader, slightly entering pronotal disc. Underside, on average, darker, with gula same coloured as lateral parts of head; anterior paler spot on elytron about 1/2 spot length distant from base, always surpassing midway between D1 and D2, often reaching or almost reaching D2 level, mostly not extending outwards beyond stria 7; posterior spots usually somewhat angulate, starting midway between D2 and D3, widely adjoining along the suture.

3rd antennomere 1.56–1.70 (mean 1.62) times as long as 2nd, 8th 2.09–2.39 (mean 2.21) times as long as wide. Pronotum 1.53–1.63 (mean 1.57) times as wide as long, 1.43–1.49 (mean 1.46) times as wide as head, more convex, especially so posteriorly; basal border obsolete medially; mid-line deeper, much so basad, adjoining transverse basal depression, latter conspicuously separated from disc convexity. Reflexed side margin adjoining disc convexity directly. Elytra 1.32–1.42 (mean 1.35) times as long as wide, 1.49–1.54 (mean 1.52) times as wide as pronotum, with base straight and transverse, as well as apices contiguous and transversely truncated at tip. Elytral striae a little deeper, resulting in intervals more convex basally and towards side margin, these 2 to 4 distinctly broader than 5 to 8 at and slightly behind the middle. Cross-striation, especially in basal 1/2 elytra, less regular, being in part transformed into deep transverse punctures. D1/EL=0.13–0.16 (mean 0.15), D2/EL=0.43–0.51 (mean 0.47), D3/EL=0.90–0.97 (mean 0.92). Metepisternum 1.47–1.55 times as long as wide.

Male profemur without ventral tubercle.

Penis (Figs 18, 27, 36) with a very coarse microsculpture and left margin in dorsal view sinuate behind basal third. Apical orifice rounded but for a distinctly sclerotized dorsolateral projection (lobe), latter being much smaller and less strongly sclerotized than in Amphimenes rugulipennis. Apical lamella rather large, strongly bent upwards and entirely occupied by a membranous window on right dorsal side.

#### Material.

Holotype ♂ (ZMMU) labelled: “S[outh] Vietnam, N[orthermost part of] Dongnai Pr[ovince]. / Nam Cat Tien Nat[ional]. Park / Exped[ition]. [of the] Russ.-Vietnamese / Tropical Centre / 22–23.X.2004 / leg. D. Fedorenko” [typewritten] “HOLOTYPE / …” [red typewritten]. Paratypes (SIEE), ♂, 4♀♀, same data but 21–22. or 28–29.XI.2004, or 18.VI.2005.

#### Type locality:

South Vietnam, Dongnai Province, Nam Cat Tien National Park.

#### Geographic distribution.

Known from type locality only.

## The *piceolus*-group

Different from the *rugulipennis*-group in the following characters: Body dorsum monochromous dark, except for paler reflexed side margins of both pronotum and elytra; these margins, as well as body appendages, less contrastingly paler. Body slenderer (PW/PL=1.30–1.55; EL/EW=1.39–1.50, being 1.33 in Amphimenes asahinai only). D1 and D2 being in posterior position (D1/EL=022–0.27, D2/EL=0.63–0.72). Wings full in all members but vestigial in Amphimenes asahinai. Dorsum without traceable cilia. Eyes large and almost hemispherical to slightly reduced and flattened, posterior supraorbital seta situated level to or slightly behind eye back margin.

The group includes six species, as follows: Amphimenes piceolus, Amphimenes asahinai, Amphimenes ryukyuensis, Amphimenes bidoupensis sp. n., Amphimenes montanus sp. n. and Amphimenes gracilis sp. n.

### 3. Amphimenes bidoupensis

Fedorenko sp. n.

urn:lsid:zoobank.org:act:16131F3A-74E4-405C-8059-E5C57693BB6B

Figs 6 19 28 37 

#### Description.

Body length 7.1–8.3/6.7–8.0 mm, width 2.7–3.1 mm. Dorsum black or dark brown, head and pronotum often a little paler, brown to reddish-brown; reflexed side margins of both pronotum and elytra, legs, mouthparts, labrum, clypeus and antennae red; femora and tibiae often slightly infuscate at middle, especially so ventrally. Underside reddish, abdominal sternites widely darkened along their posterior margins.

Eyes large, tempora short and rather abruptly extending into neck; posterior supraorbital seta situated level to eye back margin. Antennae long and surpassing pronotal base at least by last two joints, 3rd antennomere 1.54–1.88 (mean 1.72) times as long as 2nd, 8th 2.29–2.75 (mean 2.47) and 1.89–2.21 (mean 2.05) times as long as wide in males and females, respectively.

Pronotum 1.36–1.55 (mean 1.44) times as wide as long, 1.38–1.50 (mean 1.43) times as wide as head, similar to that of Amphimenes rugulipennis but the following characters: less convex, with less distinct basal transverse depression, broadest a little before middle; side margin slightly to rather strongly sinuate before hind angles, latter subrectangular due to base lateral parts sublinear and oblique only before posterolateral setigerous pore. Base medial part distinctly convex backwards, its border very shallow, especially so at middle. Mid-line rather shallow throughout its length, often shortly deeper where adjoining basal transverse depression; latter distinctly to weakly separated from disc convexity; lateral basal foveae weak but usually almost extended to anterior margin as very shallow depressions parallel to side margins. Paramedian foveae mostly shallow but distinct.

Elytra oval, 1.39–1.50 (mean 1.46) times as long as wide, 1.55–1.72 (mean 1.64) times as wide as pronotum, weakly rounded on sides, broadest at about middle; apical truncature slightly sinuate; elytral apices rounded narrowly and separately each. Elytral striae moderately deep throughout, intervals slightly convex in anterior half, inner four intervals almost flat behind the middle and turning back into convex before apex. Disc with a wide but very shallow depression between D2 and stria 6. D1/EL=0.22–0.27 (mean 0.25), D2/EL=0.63–0.72 (mean 0.69), D3/EL=0.90–0.94 (mean 0.92). Metepisternum 1.55–1.67 times as long as wide.

Last tarsomere often with proximal pair of ventral setae rudimentary or absent from some, rarely, all legs. Male profemur without ventral tubercle.

Penis (Figs 19, 28, 37) rather broad, almost straight in ventral view, with a moderately strong microsculpture, a rounded apical orifice and a fairly weak preapical dorsolateral carina; apical lamella subtriangular, slightly rounded apically, distinctly shifted mesad in ventral view.

**Figures 6–7.**
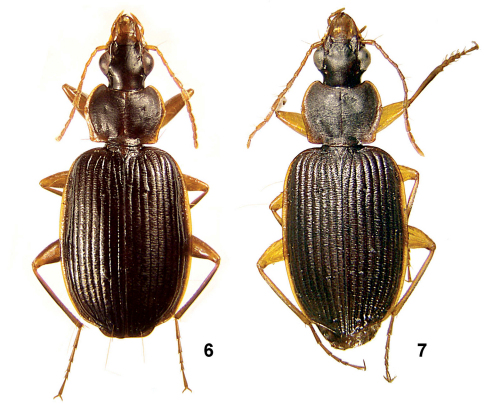
Amphimenes spp.: Amphimenes bidoupensis sp. n. (**6**) and Amphimenes gracilis sp. n. (**7**).

#### Diagnosis.

Habitually, the present species is very similar to Amphimenes ryukyuensis, Amphimenes montanus sp. n. and, especially, Amphimenes gracilis sp. n. From the former species, it differs chiefly in the combination of the larger and stouter body, namely, shorter elytra, longer pronotum, with sides distinctly sinuate before posterior angles. The latter character, together with the pronotum more strongly narrowing backwards, the narrower reflexed side border of the pronotum, and the longer metepisternum, separates it from Amphimenes montanus. The larger body and the wider pronotum, combined with the longer metepisternum, as well as certain peculiarities of penial structure, differentiate the present species from Amphimenes gracilis.

#### Material.

Holotype ♂ (ZMMU) labelled: “S[outh] **Vietnam**, Lam Dong Prov. / Bi Doup – Nui Ba Nat[ure]. Res[erve]. / env. Long Lanh / 12°10'44"N 108°40'44"E / h=1400–1600 m [a s l] / 27–28.III.2008 / leg. D. Fedorenko” [typewrittten] “HOLOTYPE / …” [red typewritten]. Paratypes (ZMMU, ZISP, SIEE): ♀, same data; 8 ♂♂, 5 ♀♀, same data but 1–2.IV.2008 or 27–28.IV., or 4.V.2009; 4♂♂, 2♀♀, same data but at light, 21–23.IV.2008; 29 ♂♂, 18 ♀♀, 12°11'N 108°42'E, ~4 km SSE of Mt Hon Giao, h=1500–1800 m [a s l], 2–3. and 7–8.IV.2008, 5. and 8.V.2009.

Other material: 2♂♂, ♀ (ZISP), **Vietnam**, Gia Lai Prov., Ka Bang distr, Krong Pa vill., 1500 m [a s l], 13–30.IX.1997 (N L Orlov); ♂, 2♀♀ (ZISP), Kontum Prov., Buonloi, 25.II.1988, 3.XI.1993 and 4.IV.1995 (Gorokhov); ♀ (ZISP), Quang Binh Prov., mountains W of Dong Hoi, “*Rao Te*” [as transcribed from Russian], 27.III.1963 (O N Kabakov).

#### Type locality:

Vietnam, Lam Dong Province, Bi Doup – Nui Ba Nature Reserve, 12°10'44"N 108°40'44"E.

#### Geographic distribution.

Widespread in montane parts of central Vietnam, from Quang Binh Province in the north to the Dalat Plateau in the south, at ~1400–1750 m a s l.

#### Life history.

The species is very common all over its type locality, where it occurs under the exfoliating bark of standing dead trees and, in addition, it flights to light at night.

#### Name.

The species name is derived from the species’ type locality.

### 4. Amphimenes gracilis

Fedorenko sp. n.

urn:lsid:zoobank.org:act:20B09C47-FD2D-4254-A14F-9449C4E56315

Figs 7 21 30 39 

#### Description.

Almost identical to the previous species, except for as follows. Body, on average, smaller, 6.4–7.5/6.2–7.3 mm long, 2.6–3.0 mm wide. Legs and antennae paler, reddish-yellow. Cross-striated sculpture denser and coarser, especially so at elytral base. Pronotum narrower, 1.29–1.42 (mean 1.37) times as wide as long, 1.35–1.50 (mean 1.40) times as wide as head. Elytra 1.40–1.50 (mean 1.45) times as long as wide, 1.56–1.72 (mean 1.66) times as wide as pronotum. Elytral striae deeper, inner four intervals more or less convex behind the middle; disc without wide and shallow depression between D2 and stria 6. Metepisternum shorter, 1.35–1.43 times as long as wide.

Penis (Figs 21, 30, 39) similar to those of Amphimenes bidoupensis and especially Amphimenes montanus both in shape and structure, but a little more robust, with apical lamella much shorter, as well as dorsolateral carina longer and much stronger, especially so basally where it forms a prominent knob.

#### Material.

Holotype ♂ (ZISP) labelled: “N **Vietnam** 40 km / NE of Thai Nguen 20 XII 1962 / leg. O N Kabakov” [handwritten] “HOLOTYPE/…” [red typewritten]. Paratypes (ZISP, SIEE), ♀, same data but 20 XI 1962 [handwritten]; ♂, 3 ♀♀, mountains 50 km / NE of Thai Nguen 300 m [a s l] / 8.2., 10.3.1963 and 16.12.1962 / Kabakov [handwritten micrographs in Russian]; ♂, ♀, [Nghe An Prov.], mountains SW [of] / Quỳ Châu 200 m [a s l] / 11.1. and 15.2.1963 Kabakov [handwritten micrographs in Russian]; ♂, S [of] Quỳ Châu ~300 m [a s l] 17.7.1963 / Kabakov [handwritten in Russian].

#### Type locality:

North Vietnam, 40 km NE of Thai Nguen.

#### Geographic distribution.

Known only from the above two localities. Occurring at lower altitudes (~200–500 m a s l.) than do Amphimenes bidoupensis or Amphimenes montanus, also probably being allopatric with both.

### 5. Amphimenes montanus

Fedorenko sp. n.

urn:lsid:zoobank.org:act:C04A0BD7-7B94-4B51-BF94-131781AB9516

Figs 8 20 29 38 

#### Description.

Body length 6.7–7.8/6.5–7.6 mm, width 2.5–3.2 mm. Same coloured as Amphimenes bidoupensis but a little darker, with femora more strongly infuscate except at apices.

Eyes prominent to slightly reduced in size and a little flattened; posterior supraorbital seta situated slightly behind eye back margin. Antennae long, 3rd antennomere 1.58–1.70 times as long as 2nd, 8th 2.0–2.22 times as long as wide.

Pronotum 1.30–1.44 times as wide as long, 1.49–1.57 times as wide as head, weakly contracted basad, with side margin almost straight between antero- and posterolateral setigerous pores, hind angles subrectangular but rounded apically. Base medial part distinctly convex backwards, with a very shallow border, especially so at middle. Mid-line rather shallow throughout and shortly deeper where adjoining transverse basal depression; latter separated from disc convexity; lateral basal foveae weak. Paramedian foveae shallow but distinct.

Elytra same as in Amphimenes bidoupensis, 1.43–1.50 times as long as wide, and 1.51–1.55 times as wide as pronotum, with no depression between D2 and stria 6. D1/EL=0.22–0.25, D2/EL=0.65–0.75, D3/EL=0.91–0.95. Mesepisternum 1.38–1.42 times as long as wide.

Last tarsomere with two pairs of ventral setae. Male profemur without ventral tubercle.

Penis (Figs 20, 29, 38) similar to that of Amphimenes bidoupensis, but apical lamella longer, dorsolateral carina less developed basally, and endophallus of different structure.

**Figures 8–9.**
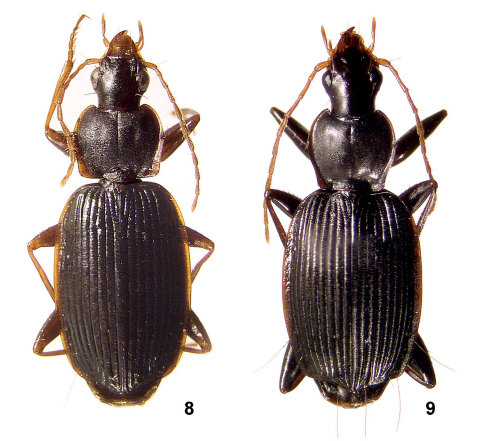
Amphimenes spp.: Amphimenes montanus sp. n. (**8**) and Amphimenes giganteus sp. n. (**7**).

#### Diagnosis.

Distinctive features see under Amphimenes bidoupensis and in the key.

#### Material.

Holotype ♀ (ZISP), labelled: “**Vietnam**, mountains / at Sa Pa, 1600–2000m [a s l]/ 8.8.1962 Kabakov” “HOLOTYPE/…” [red typewritten]. Paratypes: ♀ (ZISP), same data; ♂ (ZISP), Fansipan Mts. / 2200 m [a s l] 25.5.1963 Kabakov [all labels being handwritten micrographs in Russian]; ♂ (SIEE), “N Vietnam, Lao Cai Prov. / Hoang Lien Son Mt. Ridge / env. Fansipan Mt., Tram Ton / h=1950–2100 [m a s l] / 15–30.VII.2007 / leg. D Fedorenko.

#### Type locality:

North Vietnam, Lao Cai Province, env. Sa Pa.

#### Geographic distribution.

Known from type locality only.

## The *medius*-group

Either body colour uniform black or side margins of pronotum and elytra slightly paler; femora infuscated to dark brown entirely or except for at extremities; antennae, mouthparts, tarsi and usually also tibiae red. Antennae often slightly infuscated apicad. Eyes conspicuously to strongly reduced, posterior supraorbital setae situated far behind posterior eye margin; tempora long and smoothly extending into neck. Wings completely reduced; metepisternum very short, about as long as wide. Elytra fused along suture, oval, rather strongly rounded on sides, with strongly rounded shoulders. Discal setigerous pores on elytra either three or D1+D3; D1 and, when present, D2 being in anterior and posterior position, respectively (D1/EL=0.10–0.14, D2/EL=0.61–0.76). Setigerous pores of umbilicate series uninterrupted or weakly divided into two, posthumeral and preapical, groups. Last tarsomere mostly with one, distal, pair of ventral setae, otherwise proximal pair rudimentary. Pronotum long, only 1.13–1.29 times as wide as long, non-ciliate; medial part of pronotal base nearly straight; reflexed side margin rather narrow, especially so anteriorly.

Antennae long to very short, not reaching pronotal base. Elytral striae moderately deep throughout, intervals convex. Elytral microsculpture composed of strongly transverse meshes.

The group includes three sympatric and partly syntopic species of soil-dwelling habits.

### 6. Amphimenes giganteus

Fedorenko sp. n.

urn:lsid:zoobank.org:act:BD4D1F20-C36F-44AB-9163-F8BDC5F5124B

Figs 9 22 31 40 

#### Description.

Body length 8.5–10.6/8.3–10.2 mm, width 3.2–4.1 mm. Dorsum black, mouthparts and antennae red, clypeus and labrum mostly brown; reflexed side margin of pronotum dark brown to reddish-brown, that of elytra translucent reddish at the very base only. Gula brownish-red. Tarsi and tibiae, latter all along or apically, as well as all trochanters and procoxa red or brownish-red. Antennae often infuscated toward apex to brownish-red.

Eyes rather small and slightly flattened, a little longer than tempora; these smoothly extending into neck in dorsal view; posterior supraorbital seta situated about 1/3 distance between eye back margin and pronotal front margin. Frontal foveae fairly deep and reaching level to eye front margin. Antennae very long, surpassing pronotal base by last three or more joints, 3rd antennomere 1.88–2.05 (mean 1.95) times as long as 2nd, 8th 2.73–3.36 (mean 3.11) times as long as wide.

Pronotum subcordate, 1.18–1.26 (mean 1.22) times as wide as long, 1.37–1.49 (mean 1.43) times as wide as head, conspicuously sinuate before hind angles, with front angles pointed and approaching neck; hind angles very obtuse and strongly sloping forward. Base medial part weakly convex backward, often almost straight, with a very shallow border not extended to lateral lobes. Disc rather flat, mid-line superficial throughout its length and weakly separated from transverse basal depression, lateral basal foveae weak, each usually almost extended to anterior margin as a very shallow submarginal depression parallel to side margin. Paramedian foveae lengthwise, very shallow to indistinct.

Elytra elliptic, 1.41–1.48 (mean 1.45) times as long as wide, 1.49–1.69 (mean 1.60) times as wide as pronotum, broadest at about middle, with shoulders strongly rounded from nearly indistinct base; apical truncature strongly sinuate between protruding outer angles and a pointed apex; latter entire or divided at the very tip. D1/EL=0.10–0.14 (mean 0.13), D2/EL=0.61–0.76 (mean 0.70), D3/EL=0.90–0.98 (mean 0.94). Three or four inner striae weaker at base, prescutellary stria very weak to obsolete. Metepisternum very short, 0.96–1.0 times as long as wide.

Proximal pair of ventral setae on last tarsomere rudimentary or absent. Male profemur without ventral tubercle.

Penis (Figs 22, 31, 40) long, narrow, weakly arcuate in lateral view and abruptly curved to the right behind the middle in dorsal view; apical lamella large, long, parallel-sided, widely rounded apically; apical orifice rounded; both ventral striae and microsculpture absent.

#### Diagnosis.

The present species is easily recognizable among the other congeners by the combination of the large and wingless body, protruding outer angles of the elytral apical truncature, peculiar formula of discal setae, and other characters specified above.

#### Material.

Holotype ♂ (ZMMU) labelled: “S[outh] **Vietnam**, Lam Dong Prov. / Bi Doup – Nui Ba [Nature] Reserve / env. Long Lanh / 12°07'N 108°39'44"E / Bi Doup Mt., N. slope / h=1700–1900 m [a s l] / 12.IV.2008 / leg. D Fedorenko” [typewritten] “HOLOTYPE/…” [red typewritten]. Paratypes (ZISP, SIEE), 9 ♂♂, 9 ♀♀, same data but: 10. and 16.IV.2008, 3. and 6.V.2009; 12°11'N 108°42'E, 4 km SSE of Hon Giao Mt., h=1500–1700 m [a s l], 2–3.IV.2008 and 29.IV.2009; 12°10'44"N 108°40'44"E, h=1400–1600 m [a s l], 30.III–21 (Fedorenko).

#### Type locality:

Vietnam, Lam Dong Province, Bi Doup Mt, 12°07'N 108°39'44"E.

#### Geographic distribution.

Known from type locality only.

#### Life history.

The species is common all over its type locality, where it occurs under fallen deadwood or in its larger open cavities; it has occasionally been found under the exfoliating bark of standing dead trees, sometimes together with Amphimenes bidoupensis.

### 7. Amphimenes medius

Fedorenko sp. n.

urn:lsid:zoobank.org:act:EC986E90-E692-4AB5-B330-01420B047325

Figs 10 23 32 41 

#### Description.

Body length 5.7–7.2/5.5–7.0 mm, width 2.2–2.9 mm. Same coloured as Amphimenes giganteus, but a little paler: dorsum black, more rarely dark brown, often with head and pronotum a little paler; clypeus and labrum brown or reddish-brown; mouthparts, antennae and legs red; underside and femora, latter except at extremities, infuscated to dark brown; reflexed side margin of pronotum brownish-red; gula reddish, epipleura brownish.

Eyes rather small and flattened, a little longer than tempora; these smoothly extending into neck in dorsal view; posterior supraorbital seta situated level to about 1/3 distance between eye back margin and pronotal front margin. Frontal foveae shallow. Antennae moderately long, surpassing pronotal base by approximately last joint only, 3rd antennomere 1.40–1.58 (mean 1.47) times as long as 2nd, 8th 1.89–2.09 (mean 1.97) and 1.86–1.98 (mean 1.91) times as long as wide in males and females, respectively.

Pronotum 1.18–1.29 (mean 1.24) times as wide as long, 1.52–1.71 (mean 1.63) times as wide as head, with front angles protruding but slightly rounded apically and remote from neck; reflexed side margin narrow, especially so anteriorly, indistinctly sinuate or straight before hind angles. Base medial part weakly convex backwards, as wide as lateral lobes, these surpassing medial part and rather strongly rounded so that hind angles almost indistinct; basal border shallow and disappearing at about middle of lateral lobe. Mid-line superficial, not or hardly deeper basad, shortly deeper where adjoining basal transverse depression, latter very short, weakly to distinctly separated from a very convex disc, lateral basal foveae weak, without forward extensions along side margin. Paramedian foveae mostly indistinct.

Elytra widely oval, 1.35–1.41 (mean 1.37) times as long as wide, 1.43–1.54 (mean 1.50) times as wide as pronotum, rather strongly to (sometimes) poorly rounded on sides, broadest at about middle, with shoulders strongly rounded starting from a straight and wide base; apical truncature hardly sinuate between a rounded outer angle and a pointed apex. Discal setigerous pores two: D1/EL=0.10–0.14 (mean 0.12), D3/EL=0.91–0.95 (mean 0.93). Two or three inner striae weaker at base. Metepisternum very short, 0.86–0.89 as long as wide.

Last tarsomere with distal pair of ventral setae only. Basal third of male profemur with a distinct but wide and obtuse ventral tubercle.

Penis (Figs 23, 32, 41) triangular in ventral view, strongly twisted to the right before apex, resulting in apical orifice of almost ventral position; apical lamella large subtriangular, with a rounded tip.

**Figures 10–11.**
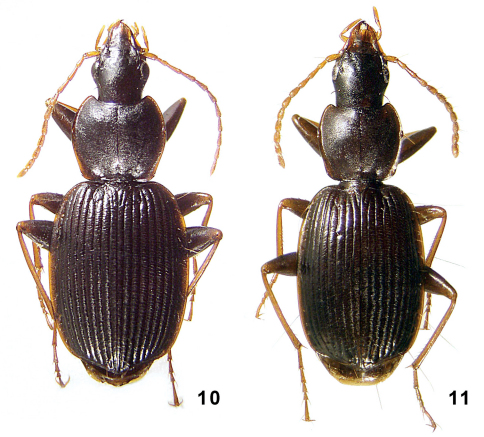
Amphimenes spp.: Amphimenes medius sp. n. (**10**) and Amphimenes minutus sp. n. (**11**).

#### Diagnosis.

Easily recognizable chiefly by the combination of the medium-sized body, D1+D3 formula of discal setae, absent wings, etc. It differs well from Amphimenes minutus in the stouter body, less strongly reduced eyes and longer antennae.

#### Material.

Holotype ♂ (ZMMU) labelled: “S[outh] **Vietnam**, Lam Dong Prov. / Bi Doup – Nui Ba [Nature] Reserve / env. Long Lanh / 12°10'44"N 108°40'44"E / h=1400–1600 m [a s l], 14–15.IV.2008 / leg. D Fedorenko” [typewritten] “HOLOTYPE/…” [red typewritten]. Paratypes (ZMMU, ZISP, SIEE), 20 ♂♂, 5 ♀♀: same data but different dates between 31.III. and 18.IV.2008, 7.V.2009; 12°11'N 108°42'E, 4 km SSE of Hon Giao Mt., h=1500–1700 m [a s l], 8.V.2009; 12°07'N 108°39'44"E, Bi Doup Mt., N. slope, h=1700–1900 m [a s l], 16.IV.2008, 3., 6. or 9.V.2009 (Fedorenko).

#### Type locality:

Vietnam, Lam Dong Province, Bi Doup – Nui Ba Nature Reserve, 12°10'44"N 108°40'44"E.

#### Geographic distribution.

Known from type locality only.

#### Life history.

Showing the same habits as Amphimenes giganteus, but never occurring under bark. The species also penetrates into rotten wood where (on Mt Bi Doup) it has been taken together with Amphimenes minutus.

### 8. Amphimenes minutus

Fedorenko sp. n.

urn:lsid:zoobank.org:act:3D264770-BEDF-4258-BE9E-58E9BBB5443D

Fig. 11 

#### Description.

Similar to the preceding species in many characters, especially body colour. The main differences are as follows:

Body small, 5.3–6.3/5.1–6.0 mm long, 2.0–2.2 mm wide. Eyes very small and flat, about as long as tempora; these very smoothly extending into neck in dorsal view; posterior supraorbital seta situated level to about midway between eye back margin and pronotal front margin. Antennae short, not reaching pronotal base; 3rd antennomere 1.38–1.45 times as long as 2nd, 8th 1.63–1.76 times as long as wide. Pronotum longer, 1.13–1.15 times as wide as long, 1.44–1.47 times as wide as head, much less convex in posterior half. Base straight, with medial part wide and almost inseparable from lateral lobes. Mid-line superficial throughout its length, basal transverse depression weakly separated from disc convexity, lateral basal foveae longitudinal and rather distinct, somewhat extended forward as very shallow depressions not reaching level of anterolateral setigerous pore. Paramedian foveae nearly indistinct. Elytra 1.42–1.43 times as long as wide, 1.50–1.55 times as wide as pronotum, with a straight but narrower base; apical truncature a little more strongly sinuate, with a slightly more protruding outer angle. Formula of discal setae seems to be D1+D3, both setae being in anterior and posterior position, respectively: D1/EL=0.12–0.14, D3/EL=0.93–0.95. Yet, two of three specimens of the type series show either an unilateral setigerous pore (“D2”/EL=0.27) situated just posterior to D1 or such an additional pore (“D2”/EL=0.33) on right elytron combined with only one anterior pore of similar position on left elytron (“D1”=0.23). Metepisternum very short, 0.9–0.97 times as long as wide.

#### Diagnosis.

The present species is easily recognizable by the combination of the small, slender and wingless body, peculiar formula of discal setae, strongly reduced eyes and short antennae.

#### Material.

Holotype ♀ (ZMMU) labelled: “S[outh] **Vietnam**, Lam Dong Prov. / Bi Doup – Nui Ba Nat[ure]. Res[erve]. / 12°07'N 108°39'44"E / Bi Doup Mt., N. slope / h=1700–1900 m [a s l], 6.V.2009 / leg. D Fedorenko” [typewritten] “HOLOTYPE/…” [red typewritten]. Paratypes (SIEE), 2 ♀♀, same data.

#### Type locality:

Vietnam, Lam Dong Province, Bi Doup Mt, 12°07'N 108°39'44"E.

#### Geographic distribution.

Type locality only.

#### Life history.

The species is rare and has been caught in rotten wood of a log together with Amphimenes medius.

## The *rufipes*-group

This monobasic group is unique first due to the combination of a strong isodiametric microsculpture on the entire dorsum combined with no cross-striation on elytra and a peculiar formula of elytral discal setigerous pores: D2+D3, D2 being in anterior position (D2/EL=0.47–0.49). Eyes distinctly reduced in size and flattened, posterior supraorbital seta situated level to about 1/3 distance between eye back margin and pronotal front margin. Wings completely reduced; metepisterna slightly longer than wide. Setigerous pores of umbilicate series uninterrupted. Last tarsomere with distal pair of ventral setae only. Antennae very short, shortest in the genus, not reaching pronotal base. Body appendages contrastingly paler than body dorsum.

### 9. Amphimenes rufipes

Fedorenko sp. n.

urn:lsid:zoobank.org:act:F8509F62-38C5-4042-9AAD-B3B06282B262

Fig. 12 

#### Description.

Body length 5.0/4.9 mm, width 2.0 mm. Dark brown, with forehead, reflexed side margins of both pronotum and elytra, suture and base of elytra paler, reddish; mouthparts, antennae and legs reddish-yellow; prosternum, notopleura, meso- and metaventrite, base of abdomen medially and elytral epipleura red.

Eyes rather small and flattened, a little longer than tempora; these smoothly extending into neck in dorsal view, latter broadest among those of other congeners. Frontal foveae very shallow. 3rd antennomere 1.2 times as long as 2nd, 8th 1.22 times as long as wide.

Pronotum 1.24 times as wide as long, 1.42 times as wide as head, rather strongly convex on disc, broadest before middle, distinctly sinuate before hind angles, front angles protruding; reflexed side margin rather narrow, only a little wider basad. Base medial part weakly convex backwards, much broader than lateral lobes, these slightly rounded posteriorly and increasingly oblique forward, resulting in hind angles very obtuse at apices; basal border obsolete over medial part. Mid-line shallow, hardly deeper before a superficial basal depression; lateral basal foveae indistinct, merging into reflexed side margin. Paramedian foveae represented by two pairs of almost indistinct, very small depressions before and behind middle.

Elytra widely oval, 1.36 times as long as wide, 1.36 times as wide as pronotum, rather strongly rounded on sides, broadest in apical third, with base very short and shoulders rounded; apical truncature hardly sinuate between distinct but rounded outer angle and a separately rounded apex of each elytron. D3/EL=0.89–0.93. Elytral intervals slightly convex, 6th and 7th subcarinate internally in basal half. Metepisternum very short, 0.82 times as long as wide.

**Figure 12.**
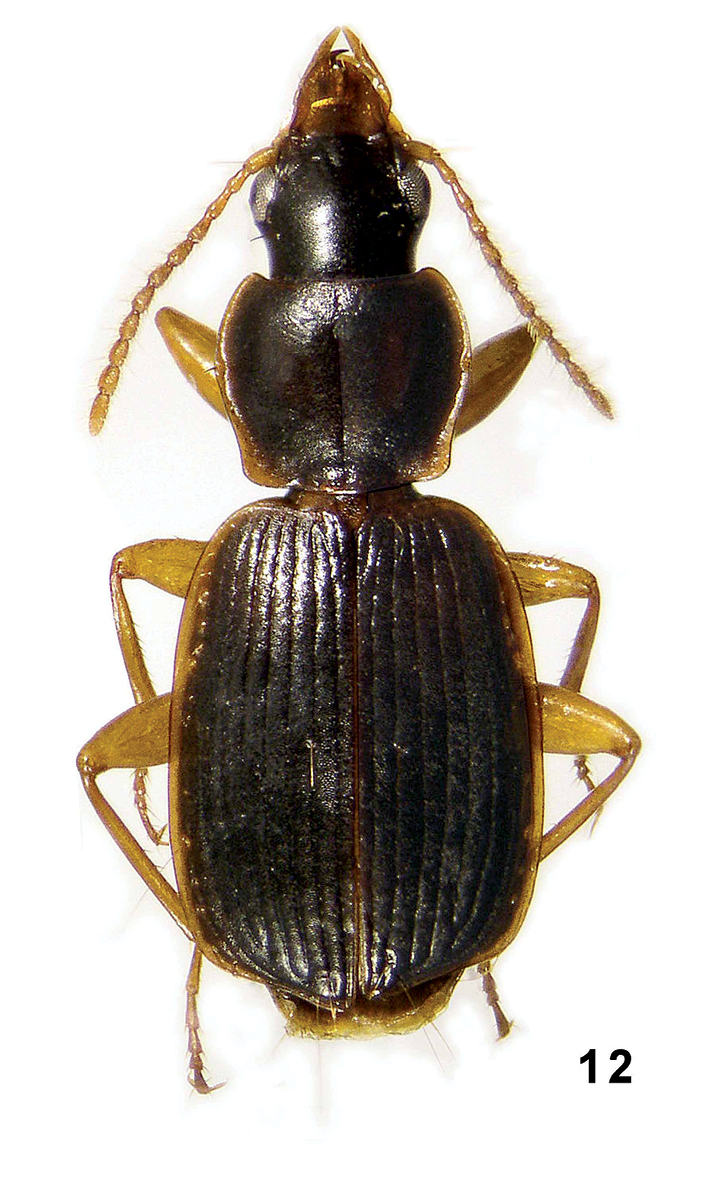
Amphimenes rufipes sp. n.

#### Material.

Holotype ♀ (ZMMU) labelled: “S[outh] **Vietnam**, Lam Dong Prov. / Bi Doup – Nui Ba Nat[ure]. Res[erve]. / 12°07'N 108°39'44"E / Bi Doup Mt., N. slope / h=1700–1900 m [a s l], 6.V.2009, leg. D Fedorenko” [typewritten] “HOLOTYPE/…” [red typewritten].

#### Type locality:

Vietnam, Lam Dong Province, Bi Doup Mt, 12°07'N 108°39'44"E.

#### Geographic distribution.

Known from type locality only.

#### Life history.

The holotype has been taken together with individuals of both previous species.

## The *planicollis*-group

Either body coloration uniform black or side margins of pronotum and elytra slightly paler; antennae and mouthparts red or reddish-yellow, either legs reddish-yellow or femora infuscated to dark brown except extremities. Eyes not or only slightly reduced in size and flattened, posterior supraorbital setae situated a little to far behind the level of eye back margin. Wings completely reduced; metepisternum about as long as wide. Elytra fused along suture, oval, rather strongly rounded on sides, with strongly rounded shoulders. Discal setigerous pores on elytra two, D2+D3, D2 situated before (D2/EL=0.41–0.45) or behind (D2/EL=0.55–0.67) middle. Setigerous pores of umbilicate series uninterrupted or weakly divided into two, posthumeral and preapical, groups. Last tarsomere mostly with distal pair of setae only, otherwise proximal pair rudimentary. Reflexed side margin of pronotum moderately to very wide posteriorly; dorsum with no cilia.

Antennae long or moderately so, surpassing pronotal base. Elytral microsculpture composed of fine and dense transverse lines or strongly transverse meshes. Cross-striation often confined to elytral base only.

The group includes four species. Of them, two, Amphimenes reflexicollis and Amphimenes planicollis, resemble and thus might have been immediate derivatives of Amphimenes montanus and Amphimenes gracilis, respectively. 

### 10. Amphimenes reflexicollis

Fedorenko sp. n.

urn:lsid:zoobank.org:act:27D515F2-DE2B-4BF3-AA51-B263715DD9EB

Figs 13 24 33 42 

#### Description.

Body length 8.5/8.3 mm, width 3.3 mm. Black, with clypeus and labrum reddish-brown, mouthparts, antennae and legs red; femora infuscated to dark brown, except at extremities; reflexed side margins of both pronotum and elytra brownish-red. Underside brown with a little paler elytral epipleura. Head and pronotum with isodiametric granulate microsculpture forming slightly transverse meshes on each side from mid-line of pronotum slightly before and behind the middle of its disc. Elytral microsculpture composed of fine and dense transverse lines, cross-striated sculpture moderately strong and developed throughout.

Eyes moderately convex, tempora rather smoothly extending into neck in dorsal view; posterior supraorbital seta situated slightly behind level of eye back margin. Frontal foveae shallow. Antennae long, surpassing pronotal base al least by last three joints, 3rd antennomere 1.9 times as long as 2nd, 8th 3.2 times as long as wide.

Pronotum rather flat, 1.39 times as wide as long, 1.56 times as wide as head, with front angles protruding, strongly and evenly rounded on sides, broadest about at middle, a little narrowing backwards, indistinctly sinuate before obtuse but very distinct hind angles; reflexed side margin wide, especially so basally; this, as well as lateral gutter distinctly though spa s ly punctulate. Base medial part about as wide as lateral lobes, these weakly rounded posteriorly, directed posterodistad and extending beyond medial part; basal border missing. Mid-line moderately deep, slightly deeper at a very shallow transverse basal depression; lateral basal foveae wide, rounded and fairly shallow, each as a shallow depression extending to about middle of pronotum parallel to its side margin. Paramedian foveae longitudinal, shallow, situated before middle.

Elytra widely oval and fairly convex, 1.47 times as long as wide, 1.52 times as wide as pronotum, broadest at about middle, with shoulders strongly rounded, apical truncature hardly sinuate between rounded posterolateral angles and very narrowly rounded, and thus almost contiguous, apices. Elytral striae deep, intervals convex. D2/EL=0.63–0.65, D3/EL=0.90–0.92. Setigerous pores of umbilicate series uninterrupted or narrowly interrupted medially. Metepisternum 1.02 times as long as wide.

Last tarsomere with two pairs of ventral setae in hind two leg pairs, but with only apical pair in fore legs. Male mesotrochanter with a weak ventral tubercle looking like a short longitudinal carina.

Penis (Figs 24, 33, 42) in ventral view almost straight and a little swollen in apical third, with apical lamella triangular and rounded at tip.

**Figures 13–14.**
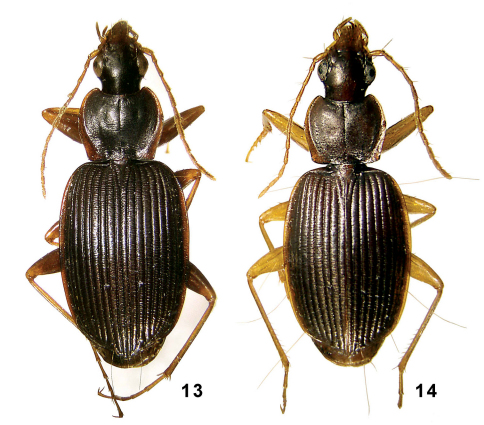
Amphimenes spp.: Amphimenes reflexicollis sp. n. (**13**) and Amphimenes planicollis sp. n. (**14**).

#### Diagnosis.

This species is easily recognizable by the combination of the fairly large body, the D2+D3 formula, the absent wings, the peculiar shape and structure of the pronotum, etc.

#### Material.

Holotype ♂ (ZISP) labelled: “**Vietnam**, Tam Dao Resort, 900 m [a s l.] / 1.7.1962 Kabakov” [handwritten micrograph in Russian] “HOLOTYPE/…” [red typewritten].

#### Type locality:

North Vietnam, Vinh Phuc province, Tam Dao.

#### Geographic distribution.

Known from type locality only.

### 11. Amphimenes planicollis

Fedorenko sp. n.

urn:lsid:zoobank.org:act:299F3A83-4EE1-490B-AADA-64B26FDDC59E

Figs 14 25 34 43 

#### Description.

Body length 5.4–6.6/5.2–6.6 mm, width 2.2–2.6 mm. Dark brown to black, with clypeus and labrum reddish, mouthparts, antennae and legs reddish-yellow; reflexed side margin of pronotum hardly paler while that of elytra distinctly so, reddish. Head and pronotum dull due to a granulate microsculpture. Elytral microsculpture composed of fine and dense transverse lines, cross-striated sculpture moderately strong and developed throughout.

Eyes slightly reduced in size and a little flattened, but tempora short and smoothly extending into neck in dorsal view; posterior supraorbital seta situated level to about 1/3 distance between eye back margin and pronotal front margin. Frontal foveae shallow. Antennae long, surpassing pronotal base al least by last 2.5 joints, 3rd antennomere 1.6–1.8 times as long as 2nd, 8th 2.5–2.8 times as long as wide.

Pronotum very flat, 1.30–1.41 times as wide as long, 1.40–1.47 times as wide as head, rather strongly rounded on sides, broadest before middle, rather strongly narrowing backwards and mostly conspicuously sinuate before hind angles, with front angles protruding; reflexed side margin moderately wide. Base almost straight, with medial part a little wider than lateral lobes, these somewhat increasingly oblique towards obtuse hind angles; basal border obsolete or absent medially. Mid-line rather shallow, slightly deeper at transverse basal depression, often almost extending to pronotal basal margin due to latter very weak; transverse basal depression angular forward but distinct only laterally where adjoining rather deep basal foveae; these extending forward into very shallow longitudinal depressions running parallel to pronotal side margin and sometimes traceable up to anterior third of pronotum. Paramedian foveae longitudinal and very shallow, ranging between missing to occupying middle third of pronotum length.

Elytra widely oval, 1.42–1.47 times as long as wide, 1.56–1.63 times as wide as pronotum, broadest at about middle, with shoulders strongly rounded, apical truncature hardly sinuate between rounded outer angles and almost contiguous apices. Elytral striae deep, intervals convex. D2/EL=0.55–0.67 (in one specimen, unilaterally 0.43), D3/EL=0.91–0.93. Setigerous pores of umbilicate series uninterrupted or narrowly interrupted medially. Metepisternum 0.8 times as long as wide.

Last tarsomere with two pairs of ventral setae. Male mesotrochanter with a small pointed ventral tubercle.

Penis (Figs 25, 34, 43) bent to the right behind middle (in dorsal view), with apical orifice secondarily extended basad.

#### Diagnosis.

This species is easily recognizable by the combination of the small body, the D2+D3 formula, the absent wings, the peculiar shape and structure of the pronotum as specified above.

#### Material.

Holotype ♂ (ZISP) labelled: “Centr. **Vietnam** / S[. of] Quỳ Châu, ~300 m [a s l.] / 17.7.1963 / Kabakov” [handwritten in Russian] “HOLOTYPE/…” [red typewritten]. Paratypes, ♀ (ZISP), same data, but 12.1.1963 [handwritten in Russian]; 2 ♂♂ (ZISP, SIEE), mountains SW of Quỳ Châu, 200 m [a s l.], 15.2.1962; 400 m [a s l.], 13. 2.1963 [handwritten micrographs in Russian].

#### Type locality:

Vietnam, Nghe An province, env. Quỳ Châu.

#### Geographic distribution.

Type locality only.

### 12. Amphimenes nitidus

Fedorenko sp. n.

urn:lsid:zoobank.org:act:38649F9F-6766-4B5D-8C71-0FCA4E33685B

Fig. 15 

#### Description.

Body length 7.5/7.2 mm, width 3.1 mm. Black, clypeus, labrum, mouthparts, antennae and legs red; femora slightly infuscated at middle; reflexed side margin of both pronotum and elytra a little paler, reddish-brown. Head and pronotum shining in spite of a coarse isodiametric microsculpture; this transformed into slightly transverse meshes before and again behind middle of disc while weakened on vertex, neck, and over a short distance along pronotal front margin on each side from mid-line. Elytral microsculpture composed of fine and dense transverse lines, cross-striated sculpture very weak and restricted to elytral base only.

Eyes slightly reduced in size and a little flattened; posterior supraorbital seta situated slightly behind eye back margin. Frontal foveae shallow. Antennae rather long, surpassing pronotal base by last two joints, 3rd antennomere 1.5 times as long as 2nd, 8th 2.3 times as long as wide.

Pronotum rather convex, especially so anteriorly, 1.54 times as wide as long and as much wider than head, with protruding front angles, slightly narrowing basad, evenly but poorly rounded on sides, broadest a little before middle, indistinctly sinuate before hind angles; side border rather widely explanate but hardly reflexed. Base almost straight, narrowly bordered, with medial part and lateral lobes subequally wide, these strongly oblique only at rounded hind angles. Mid-line rather deep throughout its length; transverse basal depression rather sharply separated from disc convexity; basal foveae small but deep and extending forward into very wide and increasingly shallow depressions, each running parallel to side margin up to middle of pronotum length. Paramedian foveae small, rounded, deep, situated before middle.

Elytra rather flat and widely oval, 1.33 times as long as wide, 1.57 times as wide as pronotum, broadest at about middle, with shoulders strongly rounded, apical truncature hardly sinuate between rounded outer angles and almost contiguous, very narrowly rounded apices. Elytral striae deep and indistinctly crenulate, intervals convex. D2/EL=0.41–0.44, D3/EL=0.91. Umbilicate series of setigerous pores uninterrupted. Metepisternum 0.9 times as long as wide.

Last tarsomere with distal pair of ventral setae only.

**Figures 15–16.**
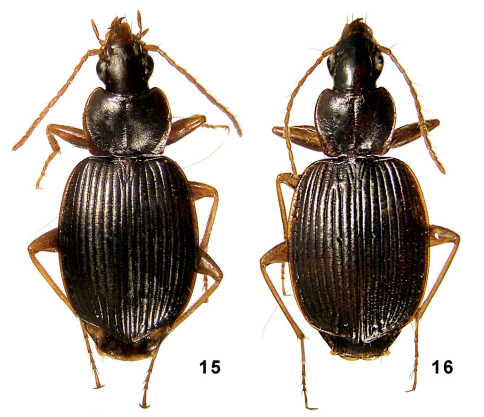
Amphimenes spp.: Amphimenes nitidus sp. n. (**15**) and Amphimenes kabakovi sp. n. (**16**).

#### Diagnosis.

This species is easily recognizable among the others by the combination of the medium-sized body, the D2+D3 formula, with D2 being in anterior position, the missing wings, the almost reduced cross-striated sculpture on the elytra, etc.

#### Material.

Holotype, ♀ (ZISP) labelled: “**Vietnam**, mountains 50 km / NE [of] Thai Nguyen, 800 m [a s l.] / 12.1.1964, Kabakov” [handwritten micrograph in Russian] “HOLOTYPE/…” [red typewritten].

#### Type locality:

North Vietnam, 50 km NE of Thai Nguen.

#### Geographic distribution.

Known from type locality only.

### 13. Amphimenes kabakovi

Fedorenko sp. n.

urn:lsid:zoobank.org:act:8DF0D22F-8C2A-4746-85E3-3539AAE60495

Fig. 16 

#### Description.

Body length 6.6/6.3 mm, width 2.75 mm. Almost black, pronotum and head dark brown, with clypeus reddish, labrum, mouthparts, antennae and legs uniform red; reflexed side margin of elytra and pronotum behind the middle reddish. Head and pronotum with a moderately deep isodiametric microsculpture resulting in both, especially pronotum, shining, latter due chiefly to a wide longitudinal band of a much weaker microsculpture occupying middle third of pronotum. Elytral microsculpture composed of strongly transverse but distinct meshes, cross-striated sculpture very weak and restricted to elytral base only.

Eyes rather strongly reduced in size and flattened; posterior supraorbital seta situated level to about 1/3 distance between eye back margin and pronotal front margin. Frontal foveae shallow. Antennae surpassing pronotal base by last 1.5 joints, 3rd antennomere 1.6 times as long as 2nd, 8th 2.2 times as long as wide.

Pronotum rather flat, 1.34 times as wide as long, 1.46 as wide as head, with protruding front angles, slightly narrowing basad, moderately widely rounded on sides, broadest before middle, indistinctly sinuate before hind angles; reflexed side border narrow, a little wider basad. Base almost straight, medially unbordered, with medial part slightly broader than lateral lobes, these very strongly oblique towards almost indistinct hind angles. Mid-line moderately deep, slightly deeper where adjoining a sharp and deep basal transverse depression; basal foveae fairly deep, weakly extending forward, externally limited by a small and flat tubercle. Paramedian foveae lengthwise, superficial, situated at middle.

Elytra rather flat, widely oval, 1.30 times as long as wide, 1.70 times as wide as pronotum, broadest at about middle, with shoulders strongly rounded, apical truncature slightly sinuate between rounded outer angles and elytral apices, these separate a little, due to their rounded tips. Elytral striae deep, intervals convex. D2/EL=0.42–0.44, D3/EL=0.91–0.92. Umbilicate series of setigerous pores uninterrupted. Metepisternum 0.9 times as long as wide.

Last tarsomere only with distal pair of ventral setae.

#### Diagnosis.

Similar to the preceding species, differing well in the smaller body, the peculiar shape of the pronotum, as well as in the meshed microsculpture of the elytra.

#### Material.

Holotype, ♀ (ZISP), labelled: “**Vietnam**, NW [of] Mt. Ridge / Tam Dao Son Zuong / 300 m [a s l.] / 20.2.1962 / Kabakov” [handwritten micrograph in Russian] “HOLOTYPE/…” [red typewritten].

#### Type locality:

North Vietnam, Tam Dao Son Zuong (Mt. Ridge).

#### Geographic distribution.

Known from type locality only.

#### Name.

The species is named in the memory of the late O. N. Kabakov, an outstanding geologist and coleopterologist from St. Petersburg, who, in addition, collected the holotype.

**Figures 17–21.**
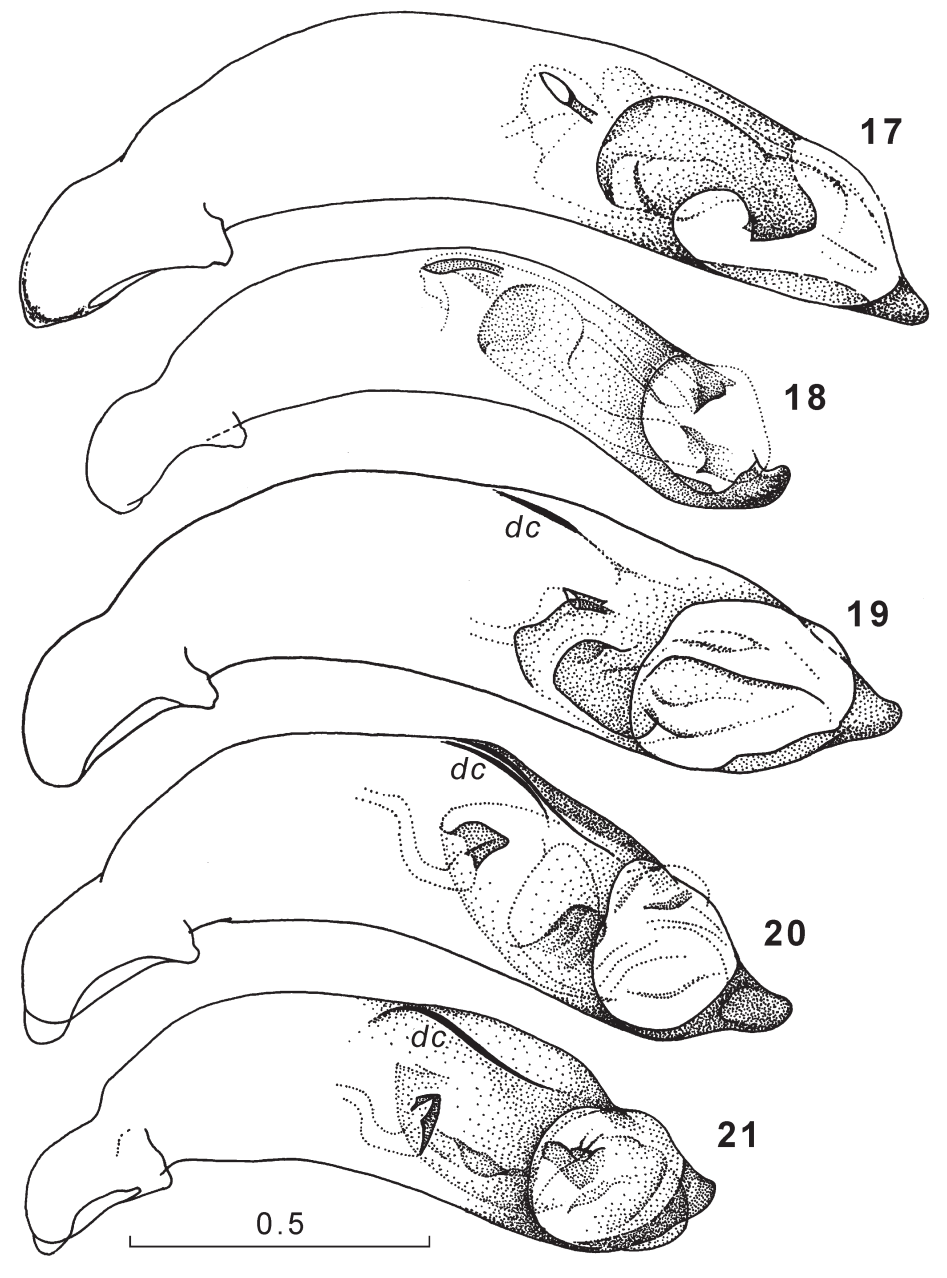
Genus Amphimenes, penis, left lateral aspect: Amphimenes rugulipennis (**17**), Amphimenes maculatus sp. n. (**18**), Amphimenes bidoupensis sp. n. (**19**), Amphimenes montanus sp. n. (**20**), Amphimenes gracilis sp. n. (**21**); *dc*, dorsal carina.

**Figures 22–25.**
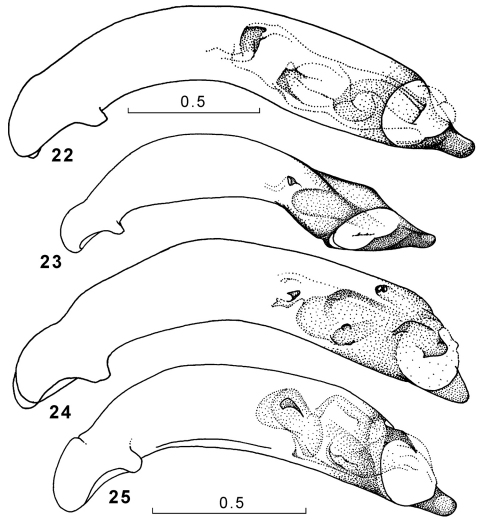
Genus Amphimenes, penis, left lateral aspect: Amphimenes giganteus sp. n. (**22**), Amphimenes medius sp. n. (**23**), Amphimenes reflexicollis sp. n. (**24**), Amphimenes planicollis sp. n. (**25**).

**Figures 26–30.**
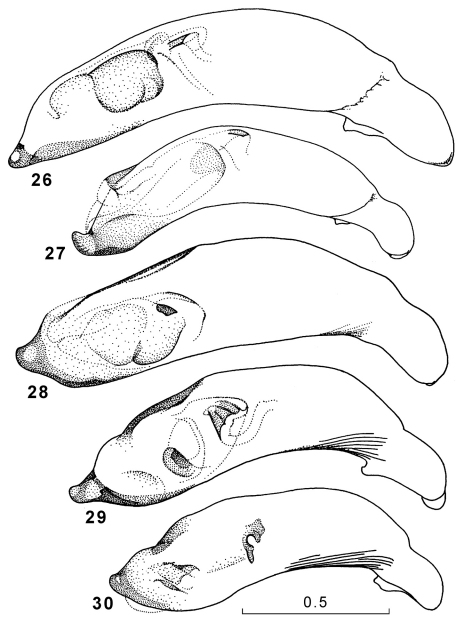
Genus Amphimenes, penis, right dorsolateral aspect: Amphimenes rugulipennis (**26**), Amphimenes maculatus sp. n. (**27**), Amphimenes bidoupensis sp. n. (**28**), Amphimenes montanus sp. n. (**29**), Amphimenes gracilis sp. n. (**30**).

**Figures 31–34.**
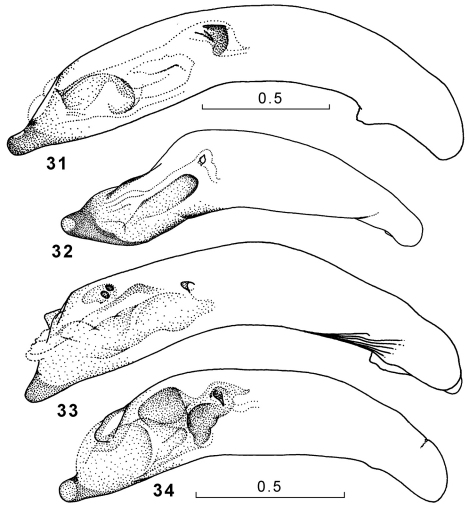
Genus Amphimenes, penis, right dorsolateral aspect: Amphimenes giganteus sp. n. (**31**), Amphimenes medius sp. n. (**32**), Amphimenes reflexicollis sp. n. (**33**), Amphimenes planicollis sp. n. (**34**).

**Figures 35–39.**
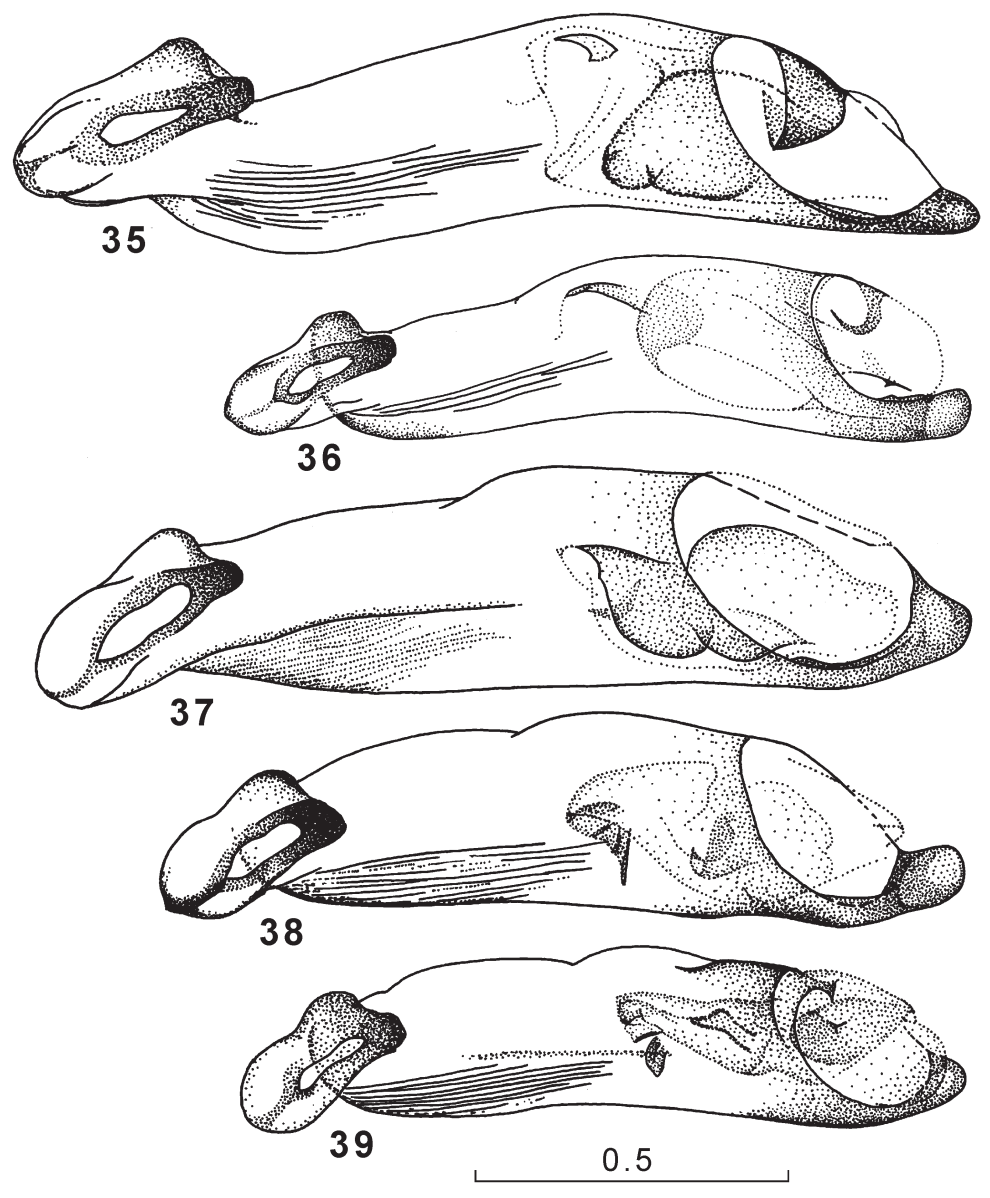
Genus Amphimenes, penis, ventral aspect: Amphimenes rugulipennis (**35**), Amphimenes maculatus sp. n. (**36**), Amphimenes bidoupensis sp. n. (**37**), Amphimenes montanus sp. n. (**38**), Amphimenes gracilis sp. n. (**39**).

**Figures 40–43.**
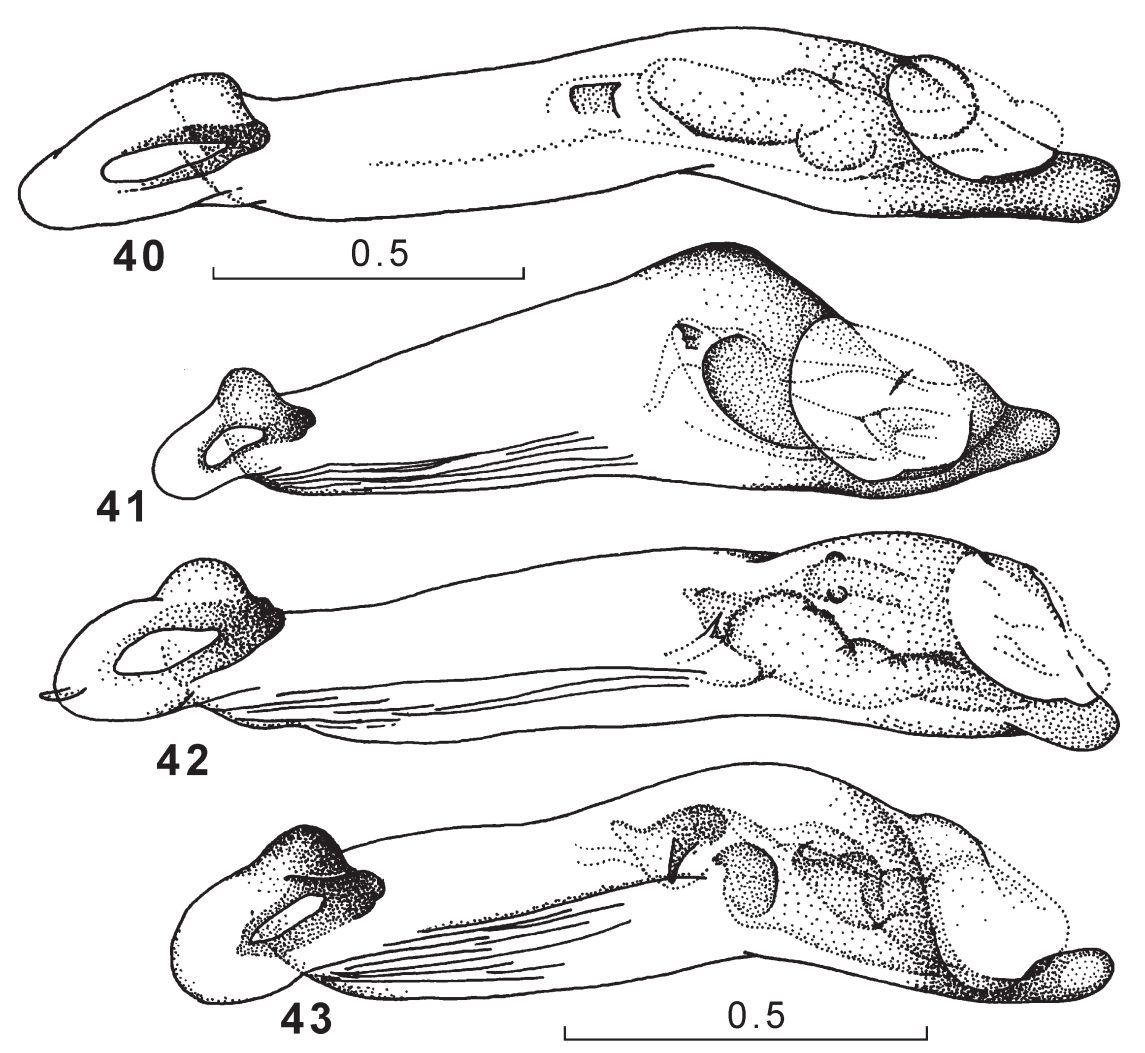
Genus Amphimenes, penis, ventral aspect: Amphimenes giganteus sp. n. (**40**), Amphimenes medius sp. n. (**41**), Amphimenes reflexicollis sp. n. (**42**), Amphimenes planicollis sp. n. (**43**).

## Supplementary Material

XML Treatment for Amphimenes

XML Treatment for Amphimenes rugulipennis

XML Treatment for Amphimenes maculatus

XML Treatment for Amphimenes bidoupensis

XML Treatment for Amphimenes gracilis

XML Treatment for Amphimenes montanus

XML Treatment for Amphimenes giganteus

XML Treatment for Amphimenes medius

XML Treatment for Amphimenes minutus

XML Treatment for Amphimenes rufipes

XML Treatment for Amphimenes reflexicollis

XML Treatment for Amphimenes planicollis

XML Treatment for Amphimenes nitidus

XML Treatment for Amphimenes kabakovi
